# Responsible Artificial Intelligence for Earth observation: human rights and the EU AI act

**DOI:** 10.1007/s43681-026-01149-5

**Published:** 2026-05-15

**Authors:** Caroline Margaux Gevaert, Sanja Šćepanović, Nadia Bernaz, Hannes Taubenböck, Mrinalini Kochupillai

**Affiliations:** 1 Faculty of Geo-Information Science and Earth Observation, University of Twente, Enschede, Netherlands; 2 Nokia Bell Labs, Cambridge, UK; 3 LAW Group, Wageningen University and Research, Wageningen, Netherlands; 4 German Remote Sensing Data Center, German Aerospace Center, Oberpfaffenhofen, Germany; 5 Institute for Geography and Geology, Julius-Maximilians-Universitat Wurzburg, Wurzburg, Germany; 6 Department of Commercial Law, Hanken School of Economics, Helsinki, Finland

**Keywords:** Human rights, Earth observation, Artificial Intelligence, Responsible, Ethics

## Abstract

The integration of Artificial Intelligence (AI) systems into Earth Observation (EO) research and innovation has catalyzed significant advancements in environmental monitoring, humanitarian response, and urban planning. However, these developments also raise novel regulatory and ethical challenges, particularly in light of the European Union’s Artificial Intelligence Act (EU AI Act), which introduces a tiered risk-based framework for the governance of AI systems. This paper provides the first comprehensive examination of how the EU AI Act, and its provisions concerning high-risk AI systems as delineated in Annex III, apply to EO-based applications. Through a structured analysis of EO use cases across key domains, such as access to public and private services, law enforcement, critical infrastructure, migration, and biometric surveillance, we illustrate how the same EO AI system may be variably classified depending on its intended purpose, autonomy level, and deployment context. We demonstrate that while many current EO AI systems are not yet autonomous enough to trigger high-risk classification, the rapid technological trajectory suggests an increasing prevalence of high-risk EO applications in the near future. Furthermore, we argue that EO researchers and developers must proactively engage with the regulatory demands of the EU AI Act, not merely to ensure compliance, but to contribute to the development of methodological tools, such as explainability, risk assessment, and auditability, that are essential for ensuring responsible AI innovation. By linking legal interpretation with technical and ethical considerations, this paper contributes to an emerging interdisciplinary framework for governing AI in the EO domain under conditions of legal uncertainty and accelerating innovation.

## Introduction

Earth observation (EO) or remote sensing research and development heavily relies on Artificial Intelligence (AI) or Machine Learning (ML) models to analyze the ever-growing number and size of EO datasets. In fact, without AI or ML, it would be either impossible or extremely time-consuming to make sense of the data, and utilize the findings and trends therefrom for real-world applications. Yet, as discussed elsewhere, e.g. [1–4], with the rapid increase in the use of AI models in EO, there is also increasing attention for the associated ethical concerns. Several of these ethical issues are now also raised, albeit in a broader context, under the EU Artificial Intelligence Act (EU AI Act).^1^ The EU AI Act seeks to regulate the marketing of systems that incorporate AI or ML models, classifies such systems on a scale ranging from “low” to “high” risk, and bans the development and deployment of certain AI applications altogether. So-called “high-risk” AI systems are regulated most heavily and are one of the main focal points of the Act.

In this paper, we provide the first comprehensive explanation of the EU AI Act’s provisions dealing with “high-risk” AI, in the context of Earth observation research and innovation. We understand Earth observation here as remotely sensed data with a perspective from above, whether from satellites, airplanes, or drones. Data recorded on the ground via camera systems is explicitly not part of the considerations here. We are of the view that our research supports the objectives of Article 6(5) of the EU AI Act which requires the European Commission to provide guidelines specifying the practical implementation of Article 6 “together with a comprehensive list of practical examples of use cases of AI Systems that are high-risk and not high-risk,” no later than February 2026. In this sense, our findings can also support recently called for evidence-based AI policy [5]. As EO research is increasingly impacting policy-making, as well as the daily lives of people, we consider this paper and its recommendations to be highly relevant and timely for EO scientists as well as policy and law makers in Europe and beyond.

### The role of ethics and human rights in Earth observation research

Under the Universal Declaration of Human Rights (UDHR) [6], both public and private entities are encouraged to respect human rights. The principles established in the UDHR can also be found in UN and regional human rights treaties, including in the Charter of Fundamental Rights of the European Union (the Charter) [7].^2^ Moreover, the 2011 UN Guiding Principles of Business and Human Rights [8] establish a corporate responsibility to respect human rights. Although non-binding, this document has contributed to a shift from considering ethical issues as part of voluntary corporate social responsibility schemes, to framing such issues as human rights ones. While ethical principles in the context of AI have not always been rigorously considered as part of human rights frameworks, they are increasingly discussed in the broader AI research community [9, 10], and this landscape has evolved with the recent enactment of the EU AI Act.

The Act aims to “promote the uptake of human-centric and trustworthy AI, while ensuring a high level of protection of health, safety, and fundamental rights enshrined in the Charter, including democracy, the rule of law and environmental protection, against the harmful effects of AI systems in the Union and supporting innovation.” It is clear, therefore, that in addition to codifying various principles of ethics, the EU AI Act also attempts to give due regard to key human rights contained in the EU Charter of Fundamental Rights. Those rights, all embedded in the Charter, are listed in Recital 48, in the introductory part of the Act.

Artificial Intelligence has been employed to interpret the vast datasets generated by EO for several decades. However, with the advent of the EU AI Act, the relevance of ethics and human rights in EO research and innovation has also become increasingly pronounced for several reasons. Firstly, as the resolution of EO images increases, privacy concerns are taking center-stage. Enhanced image resolution is also enabling the development of new EO applications that were unimaginable just a decade ago. The use of Uncrewed Aerial Vehicles (UAVs), more commonly known as drones, which can capture imagery with a ground resolution of mere centimeters, raises privacy concerns [11], capturing private areas such as backyards. Although it is possible to blur sensitive objects, research has shown that which objects would be considered sensitive varies between countries and is highly contextual [12]. A second example is the analysis of EO data, when combined with other data sources, is now being used to provide policymakers with recommendations. For example, EO applications can now spatially define which rural areas should be prioritized for electrification [13]. Similarly, EO-based slum mapping has been leveraged to localize informally populated areas [14–16] which can potentially be used to dislocate residents in preparation for international sporting events, raising significant ethical concerns [17]. The combined use of EO and other data sources, such as (social) media data, census data, among others is confounding the ethical issues [18, 19], not least because each data source comes with its own limitations and ethical caveats. While the use of EO data and AI has great potential, there are manifold aspects that are at best unconsidered or at worst accepted [4], thus emphasizing the need for more attention regarding how ethical aspects play a role in EO AI systems.

### Scope and methodology

**Fig. 1:**
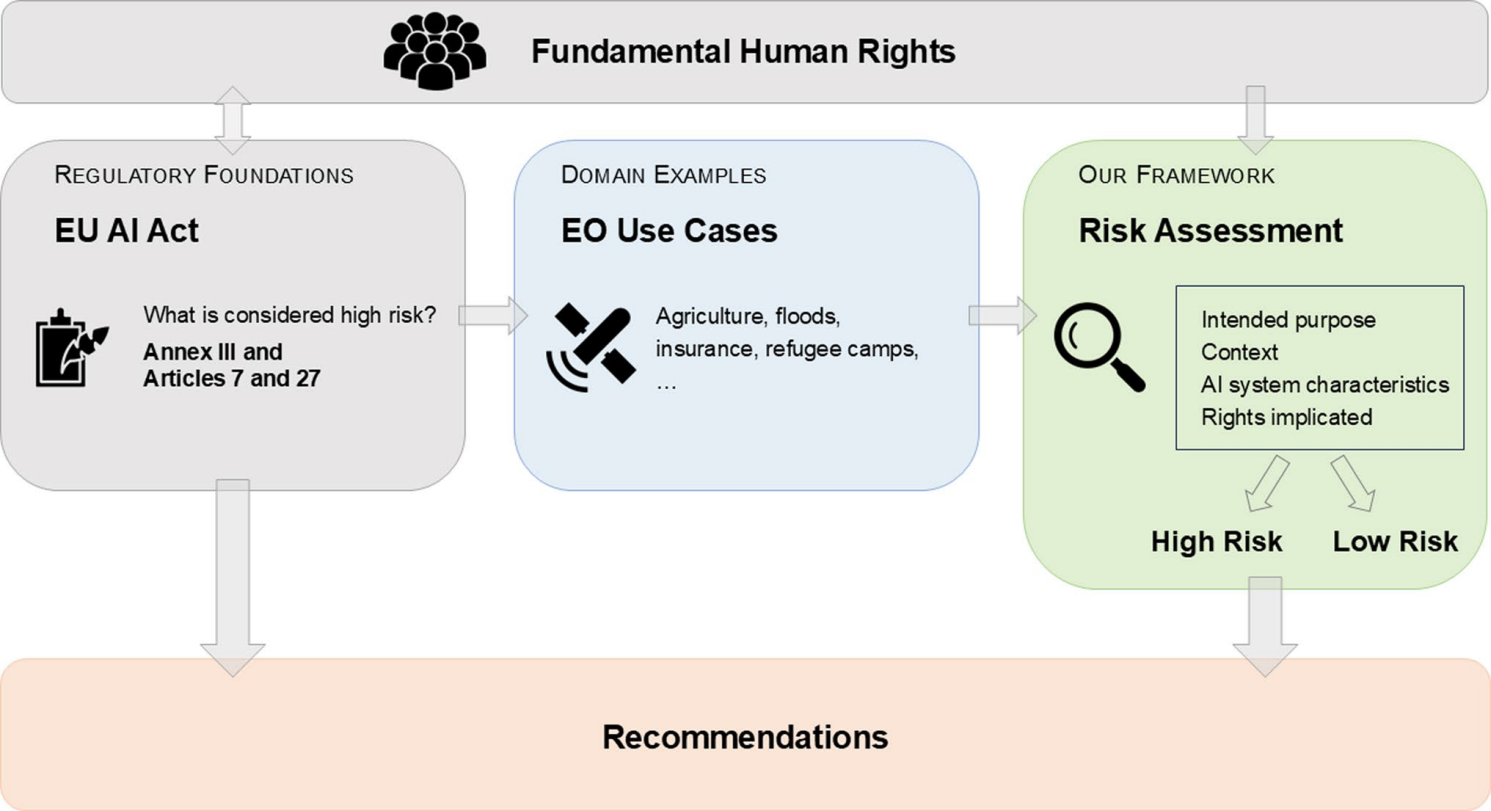
Workflow of this paper. The manuscript starts by describing relevant sections of the EU AI Act regarding high-risk AI systems (Sect. 2) and fundamental rights (Sect. 3) as shown in gray boxes. Examples of AI systems related to Earth Observation (shown in blue box) are listed in the Annex, and analyzed (Sect. 4) as shown in green box, resulting in our recommendations (red box) for the EO community and implications for policy makers in (Sect. 5)

In this paper, we contribute to the discussion on ethics, AI, and EO specifically by looking at the provisions of the EU AI Act that come into play when determining whether an AI system would be considered “high-risk” under the Act (Fig. 1). More specifically, we study Annex III, together with Sections 7 and 27 of the Act to determine how they are likely to be interpreted and applied in the context of Earth observation systems that utilize AI or ML models. An analysis of the fundamental human rights (if any) that are implicated in various EO use cases in the context of the EU AI Act is also provided. With this, we provide the first-ever guidance to EO scientists and entrepreneurs on how the EU AI Act might impact their ongoing research, development, and innovation activities. We also note that AI methods which are explicitly developed for scientific purposes, or open source systems, are excluded from the AI Act under Article 2. However, if developers or researchers are interested in some downstream exploitation activity which would involve placing these algorithms on the market or putting them into service, they would need to comply with the Act. In this sense, it is important for the scientific community to be aware of the implications of the AI Act on the topics they are researching. In addition to illustrating and analyzing EO AI systems that relate to each of the high-risk categories, to illustrate the operation of the Act, we also analyze a number of example EO use cases in more detail, using the previously mentioned Annex III and Sections 7 and 27 of the EU AI Act as the framework.

We adopt a two-sided approach, which involves (i) first understanding the provisions of Annex III of the EU AI Act with illustrations from ongoing EO research and development, and then (ii) analyzing a number of EO use cases individually under the framework derived from Annex III and Sections 7 and 27 of the Act. The latter can help the EO community analyze whether any of their ongoing EO use cases may be categorized as “high-risk” and which, if any, human rights may be impacted. The results of this detailed analysis are presented in Appendix A.

In particular, our analysis reveals that the same EO AI system may be variably classified depending on its intended purpose, autonomy level, and deployment context. We also find that while many current EO AI systems are not yet autonomous enough to trigger high-risk classification, the rapid technological trajectory suggests an increasing prevalence of high-risk EO applications in the near future.

### Structure of the paper

This paper is organized to progressively build an understanding of the EU AI Act’s implications for EO applications. Section 2 starts with an analysis of the text of the EU AI Act. It identifies the legal definition of an AI system, the criteria for an AI system to be considered “high-risk”, and regulations regarding general-purpose AI models (e.g. foundation models). Section 3 extends this analysis by examining the EU AI Act’s consideration of the Fundamental Human Rights. Thus, these two sections provide clarity on key sections of the EU AI Act that are directly relevant for developers and deployers of EO AI systems. Section 4 then transitions towards the EO domain by introducing a number of EO AI use cases. These are analyzed to assess if or under which circumstances these use cases might be considered “high-risk” by the Act. A detailed analysis for each use case is provided in Appendix A. to provide an overview of major strengths, weaknesses, and ambiguities in the EU AI Act vis-a-vis their potential impact on Earth observation research and development in the present and the future. Section 5 discusses challenges regarding the interpretation of the EU AI Act, highlights the conflicts between EU-centric Rights versus Universal Human Rights, and identifies future research directions for the EO community that will be important given the scope and wording of the EU AI Act. Finally, Sect. 6 concludes the manuscript with the main takeaways for the EO community.

## AI act and “high-risk” AI

### Background and context

To start, let us take a closer look at the relevant provisions of the AI Act. While interpreting the AI Act, it is useful to remember that it is aimed at promoting the EU-wide commercialization (“placing on the market”) of AI Systems in a harmonized and safe manner. For European enterprises, having legal certainty as to what they are permitted to do or not is an important pre-requisite to investing in and innovating with AI. The AI Act seeks to provide this legal certainty and avoid legal fragmentation so as to spur investment and innovation in AI.^3^

### Defining and understanding an “AI system”

The AI Act defines an AI system under Article 3(1), and Recital 12 details key characteristics of the definition. Article 3(1) states: an *“ ‘AI system’ is a machine-based system designed to operate with varying levels of autonomy and that may exhibit adaptiveness after deployment and that, for explicit or implicit objectives, infers, from the input it receives, how to generate outputs such as predictions, content, recommendations, or decisions that can influence physical or virtual environments”*.

Beyond this definition of an AI system, for the purpose of legal reasoning, it may become relevant to understand the difference between the “intended purpose”, “context”, “objective”, “function” and “output (generated)”. Recital 12 is of crucial relevance here because it clarifies that the “objectives of the AI system may be different from its intended purpose in a specific context.” It further explains that the environment in which an AI system operates should be understood as “context”. This “context” is different from the “outputs generated” by the AI system which reflect different functions performed by the system. For example, the function of prediction may lead an AI system to generate an output of “1” or “0” on a query as to whether a specific geographic area represents a “slum.” The objective of the system could then be “slum mapping.” However, the “intended purpose” of the system would depend on the “context” or the “environment” in which this system is deployed. For example, if the context in which the system is deployed is a congested city where it is difficult to distinguish between legal and illegal settlements, the intended purpose could be “predicting and marking unauthorized settlements for potential legal or political action.” In this specific context, it is possible or even likely that the identification of an area as a slum will lead to an entire set of people being displaced from their homes overnight; a potential violation of fundamental rights. On the contrary, if the context is that of a large developing city with limited capabilities for urban structure tracking, the intended purpose could be “transforming invisible settlements into recognized communities for service delivery improvements”. In this context, the AI System would result in improved healthcare access, infrastructure planning, and emergency response; hence, promoting human rights of the inhabitants.

Recital 12 further clarifies that the definition of an AI System in the Act aims to “facilitate international convergence and wide acceptance, while providing the flexibility to accommodate the rapid technological developments in the field”. This suggests that the Act seeks to be forward looking and the definition is likely to be interpreted broadly, so long as the AI system under consideration can be clearly distinguished from “traditional software systems or programming approaches” and does not “cover systems that are based on the rules defined solely by natural persons or automatically executed operations.” The distinguishing factor, according to Recital 12 is the AI system’s “capability to infer”. Specifically, the “capacity of an AI system to infer transcends basic data processing by enabling learning, reasoning or modelling.” In the following sections, we illustrate how modifications in the “objective”, “intended purpose”, “context” etc. can impact the assessment of whether or not an EO AI system is categorized as “high-risk.”

### AI systems considered as “high-risk” under Annex III

Chapter III of the Act goes on to list the classification rules for “high-risk AI”. Specifically, there are two broad categories of systems that can be considered “high-risk”, namely: (1) AI systems that are either themselves, or are intended to be used as, a safety component of a product under any of the Union legislations listed in Annex I; or (2) AI systems that are listed in Annex III of the Act, that have fundamental rights implications. In this paper, we focus on (2), i.e., AI systems listed in Annex III.

Annex III provides a comprehensive list of AI systems that are categorized as “high-risk”, unless they are found under Article 6(3) of the AI Act “to not pose a significant risk of harm to the health, safety or fundamental rights of natural persons, including by not materially influencing the outcome of decision making.” While Article 7 permits the enlargement of this list over time, the requirements for such enlargement are rather narrow and precise. The aim appears to be to ensure that not every AI System is inadvertently converted into a high-risk AI to the detriment of innovation and affordability of AI-based products and services in the market. Annex III comprises 8 paragraphs, each describing systems that can potentially compromise one or other fundamental human right if deployed without adequate care and human oversight (Table 1).

**Table 1:** High-risk AI system domains and associated uses as per the EU AI act

**1. Biometrics**	
Remote biometric identification systems	
Biometric categorization based on sensitive or protected attributes	
Emotion recognition	
**2. Critical infrastructure**	
AI systems to be used as safety components in the operation of critical infrastructure	
**3. Education and vocational training**	
Determine access, admission, or assignment to educational institutions	
Evaluate learning outcomes	
Assess level of education a person will receive or access	
Monitor and detect prohibited behavior of students	
**4. Employment, workers’ management and access to self-employment**	
Recruitment or selection of persons	
Decisions affecting terms of employment, including promotion or termination of contracts	
**5. Access to essential services (private or public)**	
Evaluate eligibility for public services (e.g., healthcare)	
Evaluate creditworthiness or establish credit score	
Risk assessment and pricing (e.g., life insurance)	
Classify emergency calls for dispatch or establish priority of dispatch	
**6. Law enforcement**	
Assess risk of persons becoming victims of criminal offenses	
Support law enforcement (polygraphs or similar tools)	
Evaluate reliability of evidence in investigations or prosecutions	
Assess likelihood of offending or re-offending	
Profiling of natural persons in criminal investigations	
**7. Migration, asylum and border control**	
Use by authorities as polygraphs, etc.	
Risk assessment (e.g., security, migration, health)	
Examination of asylum applications and eligibility	
Detecting, recognizing, or identifying natural persons	
**8. Administration of justice and democratic processes**	
Judicial authorities interpreting facts and applying law	
Influencing election outcomes or voting behavior	

It is notable that Annex III, when read together with Article 6, clearly seeks to avoid situations where AI is replacing or minimizing human decision-making, and therefore human responsibility, especially if such decision making implicates a fundamental human right, or the safety of people or the environment. The fact that such systems are permitted, albeit as high-risk, suggests that EU regulators are interested in checking the entry into the EU market of AI-based products and services that might replace human error of judgement with machine-based errors of judgement. The fear of AI bias based on bias in input data or other limitations of the model or data justifies the efforts to closely monitor such AI systems if they are introduced in the market. Blind replacement of human decision making by AI or machine intelligence can aggravate existing societal biases while leaving no one liable or responsible [20].

In the context of the exception under Article 6(3), it is relevant to note the clarifications provided under Recital 53 of the Act. Recital 53 details the conditions under which AI Systems will not be considered to pose “significant risk of harm” even if they fall within one of the paragraphs listed under Annex III. These conditions include AI Systems that “do not lead to a significant risk of harm to the legal interests protected under those areas because they do not materially influence the decision-making or do not harm those interests substantially,” (the terminology used here gives room for interpretation and application). Recital 53 goes on to clarify that an AI system that does not influence decision-making “should be understood to be an AI system that does not have an impact on the substance, and thereby the outcome of decision-making, whether human or automated.” This could, for example, be the case if one or more of the conditions listed in Article 6(3) and Recital 53 are fulfilled:

1. The AI system is intended to perform a narrow procedural task - e.g. the system converts unstructured data into structured data, or classifies incoming documents into categories, or helps merely to detect duplicates. Notably, however, if the categorization itself leads to the violation of a fundamental right or of a principle of trustworthy AI, it no longer remains excluded. Accordingly, for example, in the case of an AI System that automatically categorizes an incoming job application document or resume into categories that reduce the likelihood of being shortlisted, it would still be a high-risk AI under paragraph 4(a) of Annex III. In the EO context, a similar application could, for example, be the categorization of village areas influencing prioritization for electrification.
2. The AI system is intended to improve the results of a previously completed human activity, including any activity listed in Annex III. Examples could include instances where AI is used to make the language of a document more professional or improve its writing style. In the EO context, an example application could be refining human-annotated boundary delineation.
3. The AI system is intended merely to detect decision-making patterns or deviations from previous patterns. Here, the AI system is considered to be merely following a previously completed human assessment and is not replacing or influencing the assessment. New human intervention should be ensured before the identified patterns are used to make any future modifications to human decision-making. This could be the case, for example, when exam grading patterns are identified by an AI System. In the EO context, an example could be quantifying infrastructure objects in the delineated areas.
4. The AI System only performs a task that is preparatory to an assessment of whether a system falls under any of the paragraphs of Annex III. This can include, for example, indexing, searching, text or speech processing etc. or, in the EO context, image pre-processing and indexing. Whether such “preparatory” steps themselves involve a risk that might affect final human decision-making would likely need to be measured on a case-by-case basis.

If the above exceptions are found to *not* apply, an AI System falling within any one of the 8 paragraphs of Annex III is highly likely to be classified as a “high-risk” AI, and required to comply with more cumbersome regulatory checks, particularly checks under Chapter III, Sect. 2 of the Act, which include: Article 9 that requires providers to establish and maintain comprehensive risk management systems throughout the AI lifecycle; Article 10 that mandates appropriate data governance methods for collection, selection and processing; Article 11 that requires detailed technical documentation before market introduction; Article 12 that mandates automatic event logging over the system’s lifetime; Article 13 that ensures transparency by requiring provision of relevant information to deployers; Article 14 that requires human oversight mechanisms; and Article 15 that mandates high standards of accuracy, robustness and cybersecurity.

Notably, the detailed requirements under each of the above-listed articles may differ based on the “intended purpose” of the AI system, which may also need to be disclosed to the relevant authorities. As illustrated above, the intended purpose of an AI system is not merely the (mechanical or mathematical) functions the AI system performs or the output it generates. It also relates to the real world purpose,“context” and “environment” in which the system is deployed.

### Article 7 of the AI act

Article 7 discusses the conditions under which Annex III may be amended. When assessing, for the purposes of Article 7(1)(b), whether an AI system poses a risk of harm to the health and safety or a risk of adverse impact on fundamental rights that is equivalent to or greater than the risk of harm posed by the high-risk AI systems already referred to in Annex III, the Commission is required to take into account the 11 key criteria mentioned under Article 7(2), namely:

1. “Intended purpose”, which is defined under Article 3(12) as the use for which a provider designs an AI system, including specific context and conditions;
2. “Usage”, which considers how much an AI system has been or will be used;
3. “Data”, which involves examining the nature and amount of information processed;
4. “Autonomy”, which measures how independently the AI system acts and whether humans can override harmful decisions;
5. “Caused harm” which reviews actual damage to health, safety or rights already documented by authorities;
6. “Potential extent of harm” assesses the possible intensity and scope of future damage, particularly to multiple or vulnerable groups;
7. “Dependence of harm on AI system” considers how much affected persons rely on the AI’s output when opting out is impractical;
8. “Vulnerability of impacted persons” examines whether those affected are in weak positions due to status, authority, knowledge, economic circumstances or age;
9. “Outcome reversibility” determines whether the AI’s results can be easily corrected, noting that health, safety and rights impacts cannot be considered easily reversible;
10. “Magnitude of benefit” weighs the positive effects for individuals, groups or society; and
11. “Redress and risk minimisation” evaluates whether existing EU law provides effective remedies and prevention measures for AI-related risks.

### Note on general purpose AI

To finalize the analysis of the EU AI Act provisions for EO AI systems, we briefly turn to *general-purpose AI models* (GPAIs). As an example of GPAIs, foundation models (FMs) are rapidly gaining traction in EO, facilitating scalable, multimodal geospatial analysis by learning general-purpose representations across vast and heterogeneous datasets.

In the EU AI Act, a GPAI is defined as a “model is trained with a large amount of data using self-supervision at scale, that displays significant generality and is capable of competently performing a wide range of distinct tasks” (Article 3(59)). Article 51 further distinguishes between GPAIs with systemic risk, which are models with “high impact capabilities”, or those which are indicated as having systemic risk by “the Commission, ex officio or following a qualified alert from the scientific panel”. GPAIs with training above $10^{25}$ floating point operations (FLOPs) are presumed to fall under the category of models with “high impact capabilities”.

In the field of EO, recent surveys highlight an emerging class of Remote Sensing Foundation Models (RSFMs), which extend standard foundation model paradigms–such as visual FMs, vision-language models, and large language models–to address EO-specific modalities, including optical, radar, LiDAR, and textual metadata [21, 22]. These RSFMs are trained using large-scale self-supervised or multimodal pretraining to generalize across sensors, regions, and tasks. As stated before, foundation models are rapidly evolving. Numerous RSFMs are released over the past year alone–ranging from multimodal vision transformers, diffusion-based generative discriminators, to expert-mixture and temporal embedding models. For instance, IBM and ESA’s TerraMind [23] integrates nine modalities to perform tasks like semantic segmentation and land use classification, with a range of zero-shot and few-shot applications. Simliarly, the Prithvi-EO models [24] combine satellite imagery, weather, and climate data. One of the criteria for classifying a GPAI as having systemic risk is when “the cumulative amount of computation used for its training measured in floating point operations [FLOPs] is greater than $10^{25}$” (Article 51(2)). Although this information is not readily available for many RSFMs, studies report FLOPs in the range of $3*10^{11}$ for Prithvi-EO-2.0 600M models and $4.5*10^{10}$ for TerraMind 100M models to perform inference on an image [25]. The FLOPs including the training is not clear, however the order of magintude of RSFMs appears to currently be below the threshold for GPAI’s with presumed “high impact capabilities” and thus systemic risk as defined by the EU AI Act. Nevertheless, given the pace of technological change, the size of the models is likely to grow rapidly. Moreover, the EU AI Act Guidelines clarify that even if a model is under the $10^{23}$ FLOPs threshold, it could still be considered GPAI if it shows sufficient generality across many tasks. Still, the GPAI Guidelines stop short of offering a clear standard for how broad a model’s capabilities must be to qualify as sufficiently general.

Unlike high-risk AI systems listed in Annex III, GPAI are assessed for their potential to create *systemic risks*. Article 51 and Recitals 110 and 111 of the Act address these concerns: large-scale models that can be adapted for many uses may pose broad and cross-sector harms. It highlights risks such as chemical, biological, radiological and nuclear risks; disruption of critical sectors; security; and many others. There is also an understanding that the specifications of GPAI models will continuously need to be updated as technology advances. As such, the systemic risks posed by GPAIs is assessed completely differently from the high-risk classification of AI systems which should be evaluated through Annex III of the Act and that are discussed in detail in this manuscript. Thus, a separate analysis of how GPAI are considered by the AI Act would require considerable, in-depth analysis that falls outside the scope of the present paper.

## Fundamental human rights

As described in the previous section, the EU AI Act places considerable emphasis on the protection or non-violation of human rights. Recital 48 lists the rights that are most relevant in the context of the Act. We compiled these rights, as well as other rights from the EU Charter on Human Rights in Table 2.

**Table 2:** Relevant fundamental human rights (as listed in Recital 48 of the EU AI Act)

Article No	Description	Comments
Article 1	Right to human dignity	Can include right against being stigmatized, right to self-determination
Article 7	Respect for private life	Can include right against being stigmatized; drone based or, airplane or very high resolution satellite data surveillance can come into question
Article 8	Protection of personal data	Also covered under GDPR, however, this is also a qualified Human right and can be limited in some circumstances; in the context of VHR remote sensing data in combination with other geodata personalization by data combination comes into play
Article 11	Freedom of expression	Qualified right
Article 12	Freedom of assembly	Qualified right
Article 13	Freedom of art and science	Restricted under the AI Act (“responsible innovation”)
Article 14	Right to education	
Article 16	Freedom to conduct business	Restricted under the AI Act (“responsible innovation”)
Article 17(2)	Right to protection of intellectual property	Minimal disclosure needed for regulatory checks
Article 21	Non-discrimination	any localization of, e.g. places of living from VHR remote sensing data and combined with a semantic label such as slums or informal settlement, refugee shelter, large housing estate, etc. might lead to connotation of an area that might even discriminate
Article 23	Equality between men and women	
Article 24	Rights of the child	
Article 26	Integration of persons with disabilities	
Article 28	Consumer protection	
Article 31	Workers right to fair and just working conditions	
Article 37	Environmental protection, improvement of quality of the environment	the combined analysis of VHR remote sensing data with other geodata might lead to social scoring and thus to a non-equal prioritisation of measures to protect
Article 41	Right to good administration	
Article 47	Effective remedy, fair trial	
Article 48	Presumption of innocence	

Articles 13 and 16 were mentioned in the draft Act but are no longer explicitly mentioned in Recital 48

Notably, not all of the below listed human rights are “absolute”. This means that in some instances, states can restrict or take these rights away completely. In fact, it is often the case that restricting certain rights becomes necessary to meaningfully secure other rights. For example, the Freedom to conduct business (Article 16, EU Charter of Fundamental Rights) may be restricted if the business model entails encroachment of the right to protection of personal data. At the same time, the right to personal data is also not an absolute right, but a so-called “qualified right". In the EU, for example, Recital 4 of the General Data Protection Regulation (GDPR) states that “The right to protection of personal data is not an absolute right; it must be considered in relation to its functionality in society and be balanced against other fundamental rights, in accordance with the principle of proportionality.” This recital paves the way for the use of data, including personal data, in certain, albeit limited, circumstances.

Assessing whether Human Rights are potentially violated by AI systems is discussed in Article 27 of the AI Act. This article states that deployers of high risk AI systems must conduct an assessment of the impact on fundamental rights that the use of such system may produce (Article 27(1)). This assessment should include an overview of: the processes in which the high-risk AI system will be used in line with its intended purpose, the temporal scope of the system’s use, categories of natural persons likely to be affected by such use, and specific harms to these identified categories of persons, as well as an overview of human oversight measures that are implemented and which measures can be taken in case of materialization of these risks. The latter can include internal governance mechanisms and complaint mechanisms. As indicated above, this assessment should be implemented for the deployment of an AI system for a specific intended purpose and context. The assessment does not have to take place for all AI systems, but must be conducted for high-risk AI systems referred to in Article 6(2) (except high-risk AI systems intended to be used in the area listed in point 2 of Annex III.) As Article 27 stipulates, if a previous assessment exists for the same system, intended purpose and context, it is possible to use it if none of the specific aspects mentioned previously have been altered. The same article also states that the AI Office will develop a questionnaire and automated tool to faciliate deployers in complying with these obligations.

## EO AI system examples: assessing risk and human rights

To better understand the implications of the EU AI Act for EO AI systems, we adopt three strategies: First, we take the high-risk AI system categories in the EU AI Act Annex III, and identify EO applications that potentially fall into these categories. Second, we detail several EO use cases in Appendix A where we analyze in detail how the provisions of the EU AI Act could apply. Third, building on these use cases, we analyze how fundamental human rights might be implicated. This is not intended to be an exhaustive overview of fundamental rights affected by EO systems, but rather an indication of the potential applicability of the EU AI Act to EO AI systems that are released for real world testing or deployment.

As mentioned above, to illustate the possible classification of an EO AI System as “high-risk”, we detail a number of EO use cases in Appendix A. The use cases cover: (1) monitoring agricultural activities via satellite data for loan repayment; (2) insurance companies monitoring customer homes for damage assessment and premium setting; (3) automated monitoring of parking lots; (4) flood mapping and monitoring; (5) drought monitoring for food security and health; (6) detection of threatened tree species; (7) air quality downscaling; (8) water leak detection in distribution networks; (9) mapping refugee camps; and (10) mapping informal settlements. While not an exhaustive overview, their diversity serves to help illustrate how the various provisions of the EU AI Act may apply to EO AI systems.

The detailed analyses for each of these use cases are structured as follows in Appendix A. First, an overview of the case study is provided. Second, it is studied under various paragraphs of Annex III of the EU AI Act to determine whether the AI system may fall under any of the high-risk areas. Third, we analyze whether this system would be classified as “high-risk” in accordance to Annex III and various sub-sections of Article 7 of the EU AI Act. In this analysis, we provide examples of a red flag scenario that would likely be considered a high-risk use case.We also provide examples of how alterations to the objective, context, output, intended purpose, or degree of autonomy might lower this risk. Fourth, the relation to Fundamental Rights is described using various sub-sections of Article 27 of the EU AI Act. Ambiguities, if any, in the wording of the AI Act that made the classification difficult or unclear are highlighted and explained in context. When a case study involved the potential infringement of more than one human right, all were listed and analysed to determine whether the implementation of the case study involved conflicting human rights. These were flagged and the conflict explained for each use case.

### EO AI systems in high-risk domains

First, let us consider EO AI systems that could potentially fall into the “high-risk” domains of the EU AI Act Annex III. Of the eight paragraphs of high-risk AI systems in the Annex, seven appear to be relevant for AI systems related to Earth Observation (Table 3). We discuss them here, roughly ordering the paragraphs from those with stronger relevance to Earth Observation to those that are likely to have only marginal applicability. It is expected that in the context of EO, Paragraph 5 on “Access to and enjoyment of essential private services and essential public services and benefits” will be particularly relevant.

**Table 3:** Illustrative EO applications that may engage Annex III categories, depending on intended purpose and deployment context

No	AI act high-risk domain	EO applications
1	Biometrics	Facial recognition from drones (1a)
2	Critical infrastructure	Water leak detection in urban networks (2); InSAR-based monitoring of power stations and pipelines (2); Vegetation growth monitoring along powerlines (2)
3	Education and vocational training	Planning new school locations in underserved areas (3a)
4	Employment and worker management	[no convincing studies found]
5	Access to essential services	Satellite-based crop monitoring for loan repayment (5); Insurance damage assessment and premium setting (5b); Flood and drought mapping for aid distribution and eligibility decisions (5a); Informal settlement detection to guide service allocation (5a)
6	Law enforcement	Drone-based detection of violent individuals (6a, 6d); Surveillance of COVID-19 rule violations using drones (6e); Detection of illegal mining activities (6c)
7	Migration and border control	Detection of irregular activity at borders (7d); Detection of suspicious shipping patterns linked to illegal migration (7b); Mapping of refugee camps (7b, 7d)
8	Administration of justice	Damage detection in civilian buildings to support war crime documentation (8a)

Point 5(a) on the eligibility of natural persons for public assistance benefits and services can be related to the planning of services based on location information. For example, imagine an AI system that automatically detects and delivers aid to vulnerable households affected by flooding. After hurricane Ian hit Florida, the non-profit organization, GiveDirectly, used a system which detected damage using an EO AI system and overlaid the damage maps with government data on poverty to identify vulnerable persons within damaged areas. Vulnerable persons who were registered in a trusted food-stamp app received notification that they qualified for a benefit and easily enabled them to enroll for a request for a cash transfer [26, 27]. EO based research, however, has shown that financially underprivileged sections of society, the most vulnerable to increased mortality and morbidity risks from power losses, shouldered the longest outages [28]. This reveals that though EO systems deal with images of the Earth’s surface, these images capture patterns of social inequality and may convey such biases to AI system outputs and subsequent decision-making processes with unknown consequences. As will be discussed below, the intended purpose thus heavily influences whether these case studies would be considered “high-risk”.

Point 5(b) relates to evaluating creditworthiness. Monitoring agricultural activities through satellite data to assess creditworthiness is an example of such an application. The ICICI Bank in India announced the use of EO data to track agricultural practices and combine it with “demographic and financial parameters” to assess the eligibility of farmers for loans [29]. Although this is just one example, there is evidence of academic research relating EO-derived vegetation indices with credit risk (e.g., [30]).

Paragraph 2 of Annex III of the Act is also relevant for AI systems which are involved as safety components for the management and operation of critical infrastructure. There are many examples of using EO and AI systems to monitor critical infrastructure. This can be to automatically identify water leaks [31–34], using InSAR to monitor power stations and pipelines [35], to monitor vegetation growth along powerlines and thus mitigate wildfire risk [36–39], to monitor canals [40], among others. Recital 55 of the EU AI Act emphasizes that when such AI systems relate to safety components, e.g. “the failure or malfunctioning of such components might directly lead to risks to the physical integrity of critical infrastructure and thus to risks to health and safety of persons and property”. Systems such as the EO applications listed above clearly fit this category when used as a safety component to monitor the integrity of a system and inform maintenance operations.

Related to paragraph 3 of Annex III, on education and vocational training, there is evidence that EO and AI systems are being used to identify the location of schools [41], estimate accessibility to schools and educational institutions [42], and predicting quality characteristics of educational institutions such as connectivity to internet [43, 44] and overcrowding [45]. Although it is not the case yet, if the AI system were extended from assessing accessibility to determining access, prioritizing specific areas for placement of schools (at the exclusion of others) or admission decisions in education, it would be more likely to fall within Annex III point 3(a); by contrast, general school siting or planning is not straightforwardly captured by the current text of the Annex. Furthermore, if that system also posed significant risk of harm, notably to human rights (see Sect. 4.2 below), then it could also be considered high-risk under the AI Act.

Paragraph 6 on law enforcement is also relevant for EO-related AI systems. Predictive policing methods for identifying potential crime hotspots [46, 47] relates to 6(a) on assessing the risk of natural persons becoming a victim of criminal offences. AI systems using drones, in particular, can be related to 6(d) assessing the risk of a natural person offending. A study demonstrated that AI systems can be used to track specific persons from drone videos of crowds. Such systems are used, for example, in efforts to predict potential violence based on the movements of these persons. Benchmarks have also been released to support the development of further AI systems [48]. EO-based AI systems are also used for identifying ships [49], tracking illegal activity such as oil spills [50], identifying illegal mining [51, 52], logging [53], and monitoring looting of cultural heritage sites [54]. There are even preliminary studies assessing the use of satellite imagery for locating bodies in crime scenes [55, 56]. It needs to be noted that this list is not necessarily comprehensive.

Paragraph 1 of Annex III regarding the use of AI systems for biometrics is, in the field of EO applications, related to those in Paragraph 6. Specifically relevant for biometrics is facial recognition from drone imagery (e.g. [57]). The release of the DroneSURF Benchmark dataset which specifically aims to improve facial recognition from drone imagery indicates the growth of this field [58].

Applications that fall under Paragraph 7 on migration and border control are also in this vein. Satellite imagery is used by the European Border and Coast Guard Agency (Frontex) to identify boats carrying illegal immigrants across the Mediterranean Sea [59] and applications that use drones to detect occluded targets which are intended for search-and-rescue can be extended to border control applications [60]. Beyond this, EO data are for example used to classify border typologies [61] or to utilize EO data to quantify conflict intensity [62].

Finally, Paragraph 8(a) states that systems aiming to “assist a judicial authority in researching or interpreting the facts” are high-risk. This would include use cases that utilize AI systems and EO imagery to present evidence such as to document war crimes [63–65].

Regarding Paragraph 4 of Annex III, on access to employment, no EO-related applications were found that are likely to be categorized as high-risk under the AI Act. Although there is evidence for using EO to monitor work progress, e.g. think of construction site monitoring, monitoring of stock levels or completion of projects with satellite or drone imagery, for now it seems that applications focus on monitoring progress in general. If EO applications arise that lead to the assessing of the work of individual employees or applications that activate contractual clauses (see also the hypothetical use case described in A.3), then these applications would likely be considered high-risk under Paragraph 4.

While not an exhaustive overview, this analysis demonstrates that there are many AI systems using EO that could potentially fall into the “high-risk” category as they fall into the domains stipulated in Annex III of the EU AI Act. This categorization is clearly not straightforward and depends on a number of factors. These factors will be further analyzed in the following section.

### Further considerations for “high-risk” systems: exceptions and intended purpose

Once it is established whether an EO AI system falls into one of the categories presented in Annex III, the question is whether the system poses “significant risk of harm”, for which the four exceptions listed in Article 6(3) and Recital 58 help identify whether the system is such an exception. Of particular relevance is the exception 6(3)d which exempts systems which are only intended to perform a preparatory task to an assessment. Although the previous assessment demonstrates that science is developing methods for the high-risk use cases and newspaper articles insinuate that industry is already deploying AI systems in high-risk use cases, the “intended purpose” of the EO-based AI systems within the decision-making process is key. For example, a flood monitoring system designed to identify areas that have been flooded would likely fall under the “preparatory task” and would not pose a significant risk of harm. However, if the same system is automatically used to identify areas to be prioritized for aid, without significant human oversight, then it does qualify as a high-risk use case. Thus, identifying AI systems as high-risk is not straightforward and requires a nuanced analysis.

Obtaining a complete overview of the degree to which EO AI systems remain preparatory tasks or are directly used in automatic decision-making is difficult, as this information is often an internal process in industry and governments, and the methods are not made widely known to the public. However, given the experience of the authors and the level of accuracy currently obtained by AI EO systems, we expect that generally, the autonomy of EO AI systems is not yet at the level that they justify automatic decision-making, or even strong recommendation. The exception under Article 6(3)d is, therefore, very relevant in the given state of technology. However, at the same time, one can expect that the speed in the developments of EO and AI systems would make more automated systems more widely spread in the near future.

In Appendix A, we demonstrate such borderline cases by illustrating how a use case might be considered high-risk or not by changing details such as the “intended purpose” or “degree of automation”. Let us take use case 4 from our Appendix A as an example (Table 4). As discussed above, flood mapping and monitoring may fall into the “high-risk” category 5(a) or 5(b) in EU AI Act Annex III. However, as Table 4 indicates and was discussed previously, there are a number of factors which influence whether the AI system might be classified as “high-risk”. For example, if the objective is to predict damage and the AI system output is a decision to directly notify citizens to evacuate, this is a clear example of a “high-risk” system, especially if little or no human intervention and responsibility is envisaged before the direction to evacuate is communicated to the public. However, if the AI system aims to identify flood-prone areas to plan mitigation measures, whereby the system output is a map of flood-prone areas for experts to validate and discuss possible mitigation measures, then the risk-level of the AI system is likely to be significantly lower. Note that the actual flood-prediction AI algorithm underlying both these hypothetical systems could be identical, yet the 11 key criteria under Article 7(2) are likely to be considered highly relevant when determining whether the system is categorized as “high-risk” or not (as described in Sect. 2.4).

**Table 4:** Analysis of use case 4: Flood mapping and monitoring

Parameters for analysis	Red flag scenario	Possible changes to lower the risk
Objective	Predicting damage of upcoming flood event	Identify flood-prone areas to plan mitigation measures
Context	Broad deployment over different regions	City- or neighborhood scale
Output	Decision *e.g. send messages to citizens to evacuate*	Prediction *e.g. identify flood-prone areas*
Intended purpose	Protect people	Support preparedness for future hazard events
Degree of autonomy	Fully autonomous AI system	Flood maps are combined with other information to inform planning decisions
AI user	Governments	
AI subject	People	

This, and the other use cases detailed in Appendix A demonstrate how, for example, autonomous recommender systems are likely to be categorized as high-risk systems and will need to comply with the requirements set out in Article 6. Keeping a human-in-the-loop in the analysis of the data, or changing the output from an automatic “recommendation” to a “prediction” which is used to inform a human for a decision, may reduce the risk level in some instances, depending, once again, on the intended purpose of the system. It is important to note here that having a human-in-the-loop is not adequate to avoid a classification of “high-risk”. As shown by the growing field of human-computer interaction, human-in-the-loop approaches are not without limitations. In fact, they introduce new forms of overreliance risks that can arise from the interaction itself [66]. Much research has shown that human decision-making can over-rely on automation, e.g. *automation bias* [67]. Indeed, Article 14(4)(b) specifically states that deployers should communicate awareness of “the possible tendency of automatically relying or over-relying on the output produced by a high-risk AI system.” Although it is unclear how this can be enforced [68], declaration or clear quantification of uncertainty levels, for e.g. in the case of predictions, the percentage of uncertainty in the prediction, may be communicated to the user/deployer so they can determine whether or not to rely on the prediction or seek additional external inputs before making a final decision. Notwithstanding the clear communication of uncertainty levels, if the intended purpose of the system clearly falls within one of the Annex III categories and potentially violates human rights or risks safety/security, it may still be classified as high-risk.

### Fundamental rights and EO

**Fig. 2:**
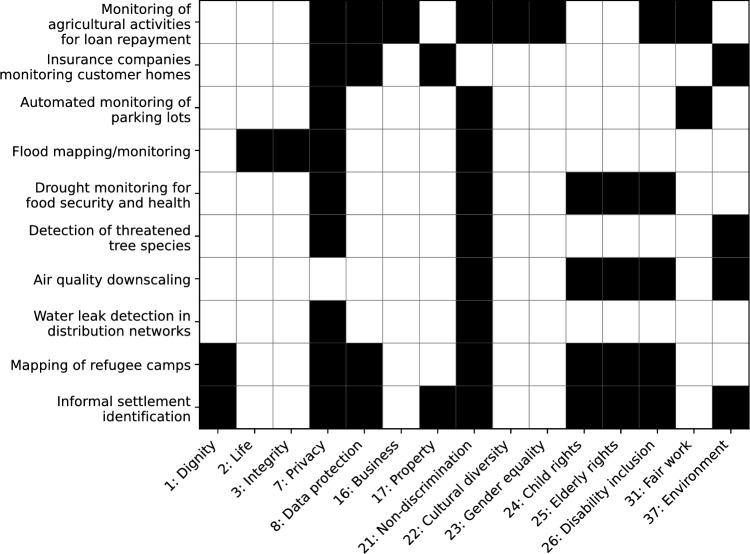
Matrix representation of fundamental human rights implicated by each EO use case. Each row lists one of our selected EO usecases, and each column (numbered according to Table 2) denotes a specific right. Filled (black) cells indicate that the right is implicated by that usecase

A second angle from which the EU AI Act analyzes AI Systems is the lens of the EU Charter of Fundamental Rights. Figure 2 presents an overview of the fundamental rights affected by the selected use cases in Appendix A. It is not intended as a complete overview, but to guide EO practitioners in understanding how fundamental rights can come into play in EO applications. Notably, even if an AI system falls under Annex III, if it does not pose a significant risk of harm to human rights, it will not be considered a high-risk AI (Article 6(3) of the EU AI Act). As a corollary, the potential risk of violation of fundamental rights is also one of the prerequisites for expanding high-risk use cases under Annex III (Article 7(1)(b) of the EU AI Act). The Article 6(3) exception has important practical implications. It means that an AI system that falls within Annex III but does not have human rights implications will not be considered high risk. As a result, EO practitioners may face situations that raise ethical concerns with which they have to deal, but which are not captured by the AI Act.

In general, Articles 7 (Respect for private and family life) and 21 (Non-discrimination) of the EU Charter for Fundamental Rights were affected in several of the use cases we analyzed, and are likely to be more broadly relevant for EO AI systems [4]. Article 7 of the Charter on Fundamental Rights deals with the right to respect for private and family life, and may be engaged every time someone can be identified or categorized using an AI system. However, Article 7 of the EU Charter and other human rights may or may not come into question depending on the expected output and the associated intended purpose of the AI system.

For example, use case 3 (see Appendix A) concerns the automated monitoring of parking lots. If the system only counts cars, then Article 7 will not come into play. If, however, the system identifies “old” cars and draws conclusions on the social situation of the drivers, then Article 7 may come into play, depending on the final output and intended purpose of the system , and depending also on whether the system poses “significant risk of harm” as per Article 6(3).

Similarly, Article 21 of the Charter that deals with non-discrimination, and is, therefore, closely related to biases in AI systems. For example, consider a use case where agricultural activities are monitored via satellite data for the purpose of ensuring timely loan repayment (see Use Case 1 in Appendix A). If the AI system makes an actual decision without human intervention (see output below), thus affecting the farmers’ livelihoods, it would engage Article 21 prohibiting non-discrimination. In this case, farmers could potentially be discriminated against due to their “social origin”, their “property”, their choice of farming system, choice of crop etc. Small farmers, particularly, would likely seek loans due to a lack of means in the first place. An equitable system would therefore ensure that human decision makers consider the facts on the ground rather than blindly relying on the AI system’s output and recommendations.

Note that the identification of biases in EO AI systems can be particularly tricky, as the dimensions of discrimination are not encoded in EO data, but can be related to spatial features. For example, although a building database may not explicitly encode the wealth or socio-economic status of the persons living there, the morphological characteristics of a neighborhood (small buildings, built close together with irregular patterns) are often associated with the wealth of the persons who can afford such a house [69]. Thus, the very characteristics of the building are connected with the social status, and AI systems may discriminate households based on wealth if certain building types are not sufficiently represented in the training data.

Notably in this context, studies have shown that crop mapping algorithms can be biased against small-holder farmers [70], and some AI-generated building detection datasets show systematic differences regarding accuracy in urban versus rural areas, and according to relative wealth [71].

Other fundamental rights which were identified in our EO case studies include Article 8 (Protection of personal data), Article 24 (the rights of the child), Article 25 (The rights of the elderly), Article 26 (Integration of persons with disabilities), and Article 37 (Environmental protection). Table 2 above presents an overview of fundamental rights which were considered relevant as they were explicitly mentioned in Recital 48 of the EU AI Act. The fundamental rights we identified in our case studies largely aligned with the fundamental rights presented in this table. However, some rights mentioned in the EU AI Act were not found in our case studies. This includes articles: 11 (Freedom of expression and information), 12 (Freedom of assembly and of association), 28 (Right of collective bargaining and action), and 48 (Presumption of innocence and right of defence). Note that this does not indicate that these rights are not relevant for EO AI systems, just that they were not identified as relevant for the case studies we analyzed for this article.

On the other hand, there are a number of fundamental rights that are not explicitly mentioned in the AI Act, but may be implicated by various EO AI Systems. These include Articles 2 (Right to life), 3 (Right to integrity of the person), 16 (Freedom to conduct business), 22 (Cultural, religious and linguistic diversity), 25 (The rights of the elderly), and 31 (Fair and just working conditions). See the detailed EO case study descriptions in Appendix A for more details on where and under which conditions these could apply.

## Discussion and guidance

### Ambiguities and challenges under the EU AI act

At a macroeconomic level, there are already ongoing debates regarding whether the provisions of the Act are so cumbersome, complex, and potentially expensive, that the law will undermine innovation by Small and Medium Enterprises (SMEs).^4^ Accordingly, it has also been speculated that the Act may be giving a monetary advantage to larger corporations that can afford to hire specialist lawyers to obtain regulatory clearance for marketing. It is necessary that administrative rules developed for the implementation of the Act acknowledge that costs and time associated with conformity assessment and documentation requirements may burden SMEs. The extent of these burdens can be minimized by making regulatory compliance affordable and transparent, minimizing complexities and ambiguities.

The process must also avoid undue delays, that can seriously impact an SME. In this context, there is already a lack of clarity on the scope and meaning of various terms. Starting with the definition of an AI System itself, going into the nuanced differences between terms such as “intended purpose” versus “objective”, and even in relation to the specific disclosures requested under the Act, there are a plethora of ambiguities and uncertainties: How will the AI Systems be continuously monitored over their lifecycle once marketed? If the disclosure of test and training data sets is mandated, who will receive these and how, if at all, will they be checked for freedom from bias? How will a change/addition to the originally declared intended purpose be identified?

Recital 165 of the AI Act states that “The development of AI systems other than high-risk AI systems in accordance with the requirements of this Regulation may lead to a larger uptake of ethical and trustworthy artificial intelligence in the Union.” The legislation, therefore, also aims to promote R&D and marketing of AI systems *other than high-risk AI systems* under a kind of self-regulation that mimics the provisions of the EU AI Act.^5^

This recital distinguishes between two streams of innovation. The high-risk AI systems stream is subject to extensive mandatory provisions, including conformity assessments, risk management, quality assurance, and documentation requirements, which may significantly increase the costs of market entry. While large corporations may internalize these costs, for SMEs they can function as a de facto barrier to entering the market and to innovation. The non-high-risk AI systems stream, on the other hand, appears to be positioned as objects of regulatory encouragement. Here, innovation is fostered through voluntary self-regulation that mimics the mandatory requirements of high-risk AI, thereby promoting the diffusion of ethical and trustworthy practices without imposing prohibitive compliance costs.

Yet, it is unclear what the legal impact of adopting “voluntary codes” would be. Will adherence to a self-inflicted code of conduct provide any legal “safe harbor” in liability contexts, or will it be merely a “good to have” for the company’s reputation?^6^

Legislation that intervenes in rapidly evolving technological domains performs not only a constraining (regulatory) but also a signaling function. By identifying areas subject to strict compliance (e.g. “high-risk” AI) obligations and others left to voluntary frameworks (e.g. “low-risk” AI), regulators are also seeking to shape the direction and volume of innovation. The legal framework under the EU AI Act can, therefore, shape the EU AI innovation ecosystem by combining mandatory constraints with voluntary (self-regulation) mechanisms, sending differentiated signals to market actors regarding the desirability of particular forms of technological development. The structure and architecture of the EU AI Act suggest this.

In this sense, the question that may be asked is whether the EU AI Act is also intended as an instrument of innovation governance, simultaneously constraining certain technological pathways while stimulating others. If this is the case, does the Act expect high-risk AI systems to only be developed and deployed by large corporations?

Further, while the AI Act lists several fundamental rights within the text, there are several that are not mentioned. The rationale for the inclusion and exclusion is not clear. Further, the fact that most fundamental human rights are not “absolute”, but “qualified”, raises the important question of balancing: the EU AI Act emphasizes the protection of human rights but fails to highlight how rights (or sets of rights) that may come into conflict with one another during the contextual application of Annex III, will be addressed or treated.

### EU-centric AI act versus universal human rights risks

The EU AI Act’s primary concerns are the European market, and the rights of people living in Europe. This means that the Act is not meant to apply when AI systems are deployed outside the EU and may impact individuals and communities there. This is particularly relevant for EO applications, as the global nature of the satellite and EO data often means that the applications and deployment of the AI systems will easily extend beyond EU borders. The AI Act thus adopts an approach that is different from the one adopted in other recent risk-based regulation such as the 2024 Corporate Sustainability Due Diligence Directive [72], amended in 2026 (Directive (EU) 2026/470). The Directive provides that large companies must address their human rights impacts outside of Europe and therefore has extraterritorial implications [73]. While both texts are part of a broader framework that strengthens corporate accountability and responsible business conduct, the AI Act has a more narrow scope. Importantly, the AI Act is not the only relevant piece of legislation in the context of AI and EO. Private EO companies may have other human rights obligations outside the Act, for example, under the mentioned Directive.

Moreover, under the Act, the applicable rights are those embedded in the EU Charter for Fundamental Rights, which does not include rights that have developed in the African, the Inter-American, and the UN human rights systems, such as the right to water, the right to food, the right to a healthy environment, and indigenous peoples’ rights. By tying the AI Act to a single European human rights instrument, certain rights are overlooked, and the protection is therefore not optimal. This deficiency may be addressed by the Courts through a more expansive interpretation of what human rights are, but this still limits the value of the AI Act from the perspective of human rights protection.

### Implications for future research directions for the EO community

It has been established that AI EO use cases can have high-risk implications, which would trigger a number of documentation and compliance obligations. This includes a regularly updated risk management system (Article 9 of the AI Act), strict data governance and management practices (Article 10), detailed technical documentation (Article 11 and Annex IV), measures for human oversight (Article 14), and methods to ensure appropriate accuracy, robustness and cybersecurity (Article 15). The extensive requirement of documentation can be used to highlight research directions within the field of EO, which will be particularly relevant over the coming years.

The data governance and management requirements stipulate the importance of design choices and data collection processes. Specific to EO data, best practices regarding the division of training, validation, and testing data with regard to possible spatial auto-correlations should be considered. Some studies show that the performance of EO AI systems can be overestimated due to spatial dependencies in geospatial data [74–76] whereas other studies found contesting results [77, 78]. Further research into best practices for data sampling and collection for the unique characteristics of EO data will be important. There is furthermore a requirement that datasets used to develop models must “be relevant, and sufficiently representative” (Article 10, Article 26, and Recital 67). This hints to the need to understand what affects model generalization. There are various references to bias mitigation (e.g. Article 10(2g), Article 11 (2g),3). All in all, the holistic approach of the technical documentation in taking the entire development of the AI system, from data acquisition to model training to deployment, supports the recent interest in data-centric learning approaches [79].

The requirement for human oversight measures highlights the importance of Explainable AI methods to improve the interpretability of results and monitor model outcomes [80, 81]. The relevance of AI-computer interaction research to understand best ways to present AI outcomes to people (e.g. asking questions instead of simply offering answers [82]) also cannot be overstated. Furthermore, the requirement for cybersecurity and the iterative nature of risk assessment motivate research into adversarial attack research [83, 84] and AI system drift detection [85].

A number of the elements and research directions described here are already on the radar for the EO research community. However, the broad impact of the EU AI Act introduces a sense of urgency for robust comparison of best practices to ensure that the documentation requirements meet their “intended purpose”.

## Conclusion

This paper provides the first in-depth assessment of how the EU AI Act will be relevant for AI Systems dealing with Earth Observation. We focus on systems which are potentially considered to be high-risk as specified in Annex III of the Act. We describe use cases that (1) could clearly fall within the scope of high-risk systems under Annex III, (2) could fall within the scope of high-risk systems depending on details such as the intended purpose, and (3) are not considered as high-risk under the current version of the EU AI Act, but could pose other ethical concerns. We expect that the largest number of EO applications may be booked as “high risk” under paragraph 5 (access to services) of Annex III. However, several EO AI systems may also be categorized under paragraph 6 (law enforcement), paragraph 7 (migration and border control) and paragraph 1 (biometrics) of Annex III. Critical infrastructure (paragraph 2) and administration of justice (paragraph 8) also have some examples from EO. Categories of high-risk applications under Annex III that appear to be less relevant for the field of EO at the moment include Paragraph 4 (employment) and Paragraph 3 (education).

Our analysis illustrates the decisive role played by the concept of “intended purpose” in the assessment of an AI system’s risk level. Further, identical AI workflows might be considered as high-risk or not depending on their deployment “context” and “environment”. For example, is an AI-generated map of areas affected by flooding used to help responders plan their support efforts or used to automate insurance compensation payments? Appendix A illustrates a number of EO use cases and how differences in their “objective”, “output”, “intended purpose”, and “degree of autonomy” might affect the risk-level of the system.

While current EO AI systems generally maintain a human-in-the-loop mechanism, it is important to note that they may nonetheless be classified as “high-risk” under the Act if they implicate human rights. Further, it is technically possible to create fully automated AI EO systems that also fully automate decision-making. Such systems would completely eliminate human responsibility and generate even higher risks, leaving no one clearly liable. The Act attempts to minimize this possibility by establishing significant human-in-the-loop requirements for high-risk AI Systems. This emphasizes the importance of pro-active human scrutiny of EO AI systems and consideration of how to frame the compliance requirements stipulated in the Act for high-risk systems. Accordingly, a number of future research directions emerge, which must be pursued to support the development of reliable compliance frameworks.

It also emphasizes the urgent need for EO researchers, developers, and deployers to understand the requirements set out by the AI Act. While R&D efforts which remain within publications and lab environments are not subject to the AI Act, if these are tested or deployed in real world scenarios, or if they influence policy making impacting the lives of people or the health of the planet, they will become subject to the Act’s provisions.

There is then also the question of how the EU AI Act’s provisions will be implemented in practice. After all, there is currently no decided court case that gives us a clear example of how the EU AI Act’s provisions are likely to be applied. Given the heavily technical nature and rapidly evolving subject matter the Act is dealing with, it is important that its provisions be understood not only by legal experts, but also by technically oriented scientists. Multi-disciplinary discussions leading up to this paper revealed that this may not currently be the case. For example, the application of the EU AI Act relies heavily on the declared “intended purpose” of an AI System that is placed under regulatory scrutiny. The “intended purpose” is so significant that if this changes, the AI System has to undergo regulatory assessment all over again. In this context, while the wording of the Act, especially Recital 12 clearly distinguish between “intended purpose”, “objective”, “context”, and “output”, this distinction may not be very clear in all EO AI use cases, especially for technically oriented scientists. It is important that regulatory bodies issue guidelines explaining these terms and the differences as well as inter-relationship between them for the scientific community and the general public. Illustrations from various fields of AI application are also necessary.

This has been routinely done for decades under other regulations that deal with technical subject matter, such as the Guidelines for Examination in the European Patent Office. There are specialized guidelines, for example, for patenting software and biotech inventions which include several illustrations. As stated previously, the AI Act seeks to provide legal certainty and avoid legal fragmentation so as to spur investment and innovation in AI. If clear guidelines on how the provisions of the Act will be implemented are not issued, it will likely lead to more uncertainty than clarity.

Moving forward, it is pertinent that the EO community engages in these policy discussions through the provision of “practical examples of use cases of AI systems that are “high-risk” and not “high risk” to the European Commission. In tandem, additional research will be needed to support the development of assessment frameworks that are tailored to the specificity of EO data and applications. This could include research directions such as bias detection, auditing systems, where to include human-in-the-loop mechanisms, and a better understanding of model generalization. Given that EO AI systems are so easily (and often) deployed in countries around the world and the limited jurisdictional scope of the AI Act - it is also essential to align EO AI systems with universal human rights and other rights which might be relevant in the country of deployment. This gap is not enforced by the AI Act but is essential for responsible deployment and wider public acceptance of EO AI systems.

## Data Availability

No datasets were generated or analysed during the current study.

## Appendix A: Detailed use-case analyses

Analyzing whether a use case can be considered high-risk depends not only on the topic and objectives of the system, but also on the context, output, and most importantly, the intended purpose of the system. Of crucial importance also is the question of whether the AI system poses a *significant* risk of harm to human rights. As explained in Article 6(3), even if a system falls under Annex III, it will not be considered high risk if, for example, if does not “materially influence the outcome of decision making” To illustrate the complexities of whether a use case might be considered high-risk, we provide an extended analysis for several EO use cases.

Each analysis provides: a description; the relevant high-risk area in Annex III of the EU AI Act; a table describing the AI system according to the requirements specified in Article 7(2); and a detailed analysis of the fundamental rights that might be affected by the AI system and possible ensuing harms as outlined in Article 27. The analyses according to Article 7(2) and 27 are slightly simplified here and focus on the most relevant points to ensure conciseness. The definitions of the different components in accordance with the AI Act are listed below.

1. **Intended Purpose:** the intended purpose of the AI system, defined in Article 3(12) as “the use for which an AI system is intended by the provider, including the specific context and conditions of use, as specified in the information supplied by the provider in the instructions for use, promotional or sales materials and statements, as well as in the technical documentation”.
2. **Objective:** It is what the AI system is trained to infer (either from machine learning approaches that learn from data, or from logic- and knowledge-based approaches that infer from encoded knowledge or symbolic representation of the task to be solved). For example, an AI System can be trained to identify a cat, or a slum. So, its objective is to identify a cat or slum. An AI System operates according to implicitly or explicitly defined objective.
3. **Context:** The environment or context in which the AI system operates. Or, the domain that represents the area or sector the AI system is intended to be used in.
4. **Output:** Outputs generated by an AI system reflect different functions performed by it, e.g. predictions, content, recommendations or decisions. This output influences physical or virtual environments.
5. **Degree of Autonomy:** AI systems are designed to operate with varying levels of autonomy, meaning that they have some degree of independence of actions from human involvement and of capabilities to operate without human intervention. The higher the degree of autonomy, the greater the risk level if human rights are potentially violated. This is because with rising levels of autonomy, human responsibility and capability to pin liability on a human agency decrease.
6. **AI User:** The entity or individual in charge of deploying and managing the AI system, including individuals, organizations, corporations, public authorities, and agencies responsible for its operation and management.
7. **AI Subject:** The individual directly affected by the use of the AI system, experiencing its effects and consequences. They interact with or are impacted by the AI system’s processes, decisions, or outcomes.
8. **Harm:** Describes the human rights that are violated, referring to the EU Charter of Fundamental Rights articles and a brief description of the actual harm (actual harm e.g. economic, social, reputational, etc.). A detailed description on how these rights come into play is provided for each use case.

Note that in each of the below tables describing the AI system, we indicate a “red-flag” (high risk) scenario where a particular EO application is clearly high-risk. We also describe how certain parameters could be changed to lower the risk. Of key relevance is the “intended purpose” of the AI System and the associated outputs. Indeed if the intended purpose of an AI system changes, the risk level of the AI system must be checked again. The intended purpose is also closely linked with other system components. For example, the output of an AI system can be content, predictions, recommendations, or decisions; this list being generally ordered from lower to higher risk. Automated decisions always come with the highest risk. Note that the output will again depend on the “intended purpose” of a system. Is it to automate processes, or to inform decision-makers? In our analyses below, we indicate how changes to the “intended purpose”, “objective”, “context”, “output”, and “degree of autonomy” can alter the risk level of the AI system.

These possible changes can be made individually, and making this change does not guarantee that the system changes to low-risk. It is mainly intended to inform EO practitioners of the types of adaptations that can help lower the risk of an AI system as defined in the EU AI Act.

### A.1 Use case 1: monitoring of agricultural activities via satellite data for loan repayment

**Description:** Financial institutions may employ satellite data and analytics service providers to continuously monitor the agricultural operations of their loan recipient farmers. Specifically, banks may seek to verify timelines of crop harvesting to align with agreed-upon loan repayment schedules.

**Relevant high-risk area in Annex III of the EU AI Act:** 5(b) “Access to and enjoyment of essential private services (...)” In this example, the “essential private service” is “loan availability/eligibility” (Table 5).

**Table 5:** Analysis of use case 1: monitoring agricultural activities

Parameters for analysis	High-risk scenario	Possible changes to lower the risk
Intended purpose	Timely loan repayment enforcement	Urging local agriculture extension officers to help farmers
Objective	Trigger action for enforcing loan repayments based on harvest predictions from EO imagery OR automatically impact credit rating of farmer in case of apparent “delays” in loan repayment	Suggest when the crop is ready for harvest
Context	All farm sizes and types; Large monocultures, small farms etc	Specific types of farms, e.g. small farms only
Output	Decision *e.g. repayment is automatically demanded in two weeks*	Prediction *e.g. harvest should start in two weeks*
Degree of Autonomy	Fully autonomous AI System	Strong human in the loop requirement
AI user	Bank	–
AI subject	Farmers	–

The table above describes hypothetical examples of a high risk scenario and of a similar scenario that would actually not be high risk due to key differences. The scenario in the first coloumn is labelled as a “High-risk scenario”, first because it falls within Annex III paragraph 5(b); and second because of the following factors: (1) the intended purpose (loan repayment) can engage a number of human rights (see in-depth analysis below); (2) the objective is to enforce loan repayment or adjust farmers’ credit ratings, which can both potentially interfere with human rights (also see below); (3) the output is a decision taken by the AI system; (4) the AI system is fully autonomous. By contrast, in the second column labeled “Possible changes to lower the risk”, the intended purpose and objective do not directly impact human rights. The system does not aim to ask or take money from people or to coerce them into acting in a certain way, but to provide information that can then be used to provide help to farmers. In addition the output is a prediction, only meant to help humans make a decision themselves. In the same vein, the system is not fully autonomous. Used by humans as a form of help to make decisions that do not impact human rights, the use of the AI system is thus low risk.

***In-depth analysis of fundamental rights and harm:***

The use of AI in this example may infringe on the right of farmers to respect of their private and family life (Article 7) as well as the right to the protection of personal data (Article 8). If the AI system makes an actual decision without human intervention (see ‘Output’), thus affecting the farmers’ livelihoods, this use case also engages Article 21 prohibiting non-discrimination. In this case we can say that the farmers would be discriminated due to their “social origin” but also arguably their “property” since they would presumably seek loans due to lack of means in the first place.

Article 16 that protects the freedom to conduct a business, may also be violated. Farmers’ decisions to sell their production, a pre-condition to repaying the loans, are business decisions that should be freely taken based on, for example, trends in market prices. The use of AI to automate the decision to request loan repayment takes away this freedom. If the delay automatically reported by the AI System also leads to an automated lowering of the farmer’s credit rating impacting the farmer’s eligibility to acquire loans in the future, the system creates further risks of harm to fundamental rights.

In a similar vein, in this case, the right of farmers to fair and just working conditions (Article 31) is also potentially infringed. Once the satellite observation has revealed that harvest has started, this may trigger automated decisions to ask for loan repayment after a fixed period. However, several factors may influence the pace at which crops are harvested, particularly the availability of labor, weather conditions etc. This may lead farmers to engage in unsafe and unjust work and/or to require it from their employees.

In addition to those core rights (Articles 7, 8, 16, 21 and 31), this use case can potentially infringe other rights, depending on circumstances. Those include cultural, religious and linguistic diversity (Article 22), if for example harvest is taking longer than usual due to religious or cultural events affecting the pace and intensity of labor. In many contexts, farm work has gendered implications and is dependent on farmers’ care and family responsibilities, which can engage Article 23 on equality between men and women. The use case may also engage Article 26 on the rights of people with disabilities, particularly their right “to benefit from measures designed to ensure their independence, [and] social and occupational integration.”

Economic harm caused by forced early repayment or inequitable access to insurance, particularly for small-holder farmers or farmers choosing non-conventional agricultural techniques. If it is only about repayment, that is reversible. If, however, in the process of banks pressuring the farmers, their livelihood is endangered due for example to their particular economic vulnerability, there could be some less reversible outcomes.

### A.2 Use case 2: insurance companies monitoring customer homes for damage assessment and premium setting

**Description:** Insurance companies use satellite data to assess disaster risks like floods or earthquakes, aiding in accurate damage estimates and client repayments. This data also informs premium settings, adjusting rates based on an area’s susceptibility to natural hazards or even the quality of building materials.

**Relevant high-risk area in Annex III of the EU AI Act:** 5(b) “Access to and enjoyment of essential private services (...)” Here the “essential private service” could be access to insurance services, their affordability, and/or equitable access to insurance payouts (Table 6).

**Table 6:** Analysis of use case 2: Insurance companies monitoring customer homes

Parameters for analysis	High-risk scenario	Possible changes to lower the risk
Intended purpose	Efficient premium setting and verifying damage claims	Planning field visits to verify damage claims
Objective	Quantify damage and hazard risk	Identify areas likely damaged
Context	Broad deployment over different regions	Specific hazard event
Output	Decision *e.g. which policies should be activated and which not*	Prediction *e.g. which areas are likely to have higher damage*
Degree of autonomy	Fully autonomous AI System	Strong human in the loop requirement
AI user	Insurance companies	–
AI subject	Homes/property of natural persons	–

The table above describes hypothetical examples of a high risk scenario and of a similar scenario that would actually not be high risk due to key differences. Here the scenario is high risk first because it falls within Annex III paragraph 5(b); and second because of the following factors: (1) the intended purpose (setting premiums and verifying damage claims) can engage a number of human rights (see in-depth analysis below); (2) the objective is to quantify damage and hazard risk, which can both potentially interfere with human rights (also see below); (3) the AI system is deployed over a large area, thus is more prone to error (4) the output is a decision taken by the AI system; (5) the AI system is fully autonomous. By contrast, in the lower risk scenario, the intended purpose and objective do not engage human rights. The system does not aim to potentially increase peoples’ insurance expenses (which could lead to decrease the value of their property), but to provide information so that field visits to assess damages can be more systematically planned (e.g. by prioritizing certain areas). Deployed following a specific incident, in a smaller area, the AI system is arguably more accurate. In addition the output is a prediction, only meant to help humans make a decision themselves. In the same vein, the system is not fully autonomous. Used by humans as a form of help to make decisions that do not impact human rights, the use of the AI system is thus low risk.

***In-depth analysis of fundamental rights and harm:***

This use case engages Articles 7 and 8 due to possible privacy breaches (see use case 1 above). It may also lead to infringements of Article 21 on non-discrimination if for example specific areas are targeted. Article 17 on the right to property may also be threatened. The automated monitoring of customer homes, for example to set premiums, may impact the overall value of the properties which may increase or decrease depending on what decision is made. Arguably this may affect the right to property (Article 17) if the value of the home decreases. This use case also potentially engages Article 37 on consumer protection. This is because the affected individuals are customers of the insurance companies but also consumers of the service those companies provide.

The harm caused can be economic harm if damage is not recognized. It can also be social harm if homes in perceived high-risk areas are denied coverage. If it is only about repayment, that is likely reversible. However, this could also have long-term harms such as lowered credit scores that are difficult to reverse and particularly harmful for already economically disadvantaged people who are more vulnerable.

### A.3 Use case 3: automated monitoring of parking lots

**Description:** The COVID-19 pandemic brought attention to this application as satellite imagery revealed significant fluctuations in factory outputs, visualized through cars in parking lots. The technology has various implications, from estimating production output to potentially unearthing labor or environmental issues. Similar technology was also used for estimating how popular/busy a supermarket is at a specific time.

**Relevant high-risk area in Annex III of the EU AI Act:** 1(a–c) (Table 7)

**Table 7:** Analysis of use case 3: Monitoring parking lots

Parameters for analysis	High-risk scenario	Possible changes to lower the risk
Intended purpose	Identification of parking lot occupancy and tracking of individuals	Identification of parking lot occupancy for consumer convenience
Objective	Monitoring cars and individuals in factory or warehouse parking lots	Monitoring cars in supermarket or concert venue parking lots
Context	Industrial sector	Retail or entertainment sectors
Output	Prediction *e.g. presence of specific individuals*	Prediction *e.g. location and number of cars allow people to plan finding available parking*
Degree of autonomy	High autonomy with direct impact on workers, i.e., automated decisions affecting employment conditions	Low autonomy with human oversight, i.e, informational system requiring human interpretation or allowing human choice
AI user	Companies	Companies
AI subject	Employees	Consumers

The table above describes hypothetical examples of a high risk scenario and of a similar scenario that would actually not be high risk due to key differences. A key element in this use case is whether it is merely used to track cars in general or whether individual cars, or persons, can be identified. In the table, the scenario in the left column is high risk first because it falls within Annex III paragraph 1(a)-(c); and second because of the following factors: (1) the intended purpose (which includes tracking of individuals such as union leaders) can engage a number of human rights (see in-depth analysis below); (2) the AI system is fully autonomous with no human involved. By contrast, in the lower risk scenario, the intended purpose is to increase consumer convenience, which is in principle not problematic from a human rights perspective. In addition humans remain involved in the actual decision.

***In-depth analysis of fundamental rights and harm:***

If the system is used merely to count cars, then no right is likely engaged. However, if the system is coupled with facial recognition or the characteristics of person, or can differentiate between different “types” of people (e.g. are they white? Are they overweight? Do they wear a hijab? Is their car more than x years old and thus are they poor?), then we enter into high-risk territory (Annex III, paragraphs 1(a)–(c)). This is especially the case because such facial recognition would be used here by a private company for private purposes and not, for example, by a government for the purpose of public safety. Fundamental rights affected could be: Article 7 (private and family life), and Article 21 (non-discrimination). In addition, when monitoring employees, the right to fair and just working conditions (Article 31) can also be engaged.

By identifying individual workers or their vehicles, the AI system could infringe on their privacy rights (Article 7). If this information were to be used against them personally or were to lead to them being treated unfairly by their employers, this would be discriminatory and possibly constitute a violation of Article 21. In addition bias could be introduced if only certain factories or regions are monitored, possibly leading to discriminatory practices; revealing this data/results could influence company stocks; however, this is not the problem of the AI system itself but of its selective use in some and not all industries/regions.

In this use case, while the workers are not necessarily vulnerable as such, there is still a clear imbalance between those tracking and those being tracked, leading to vulnerability. Whether or not the outcome, for example unfair dismissal, is reversible depends on whether the work can easily appeal the decision. Here it is important to note that having the right to contest decisions before for example an employment tribunal does not necessarily mean that workers will exercise this right with ease as many factors impact access to justice (rights awareness, financial means, caring obligations, etc.). Those factors must be taken into account while considering the reversibility of outcome.

### A.4 Use case 4: flood mapping and monitoring

**Description:** EO data can be used to produce flood maps, although it would be more accurate to call them flood proxy maps, since they do not reveal the entire floods, but are often the best substitute for a map. These maps can be produced in rural areas/bare soil, but also in urban areas, with the latter being a much more sophisticated process. If the mapping activity is repeated periodically (e.g., daily), then it is called monitoring. Monitoring allows to observe the evolution of flood inundations. Several decision-making processes rely on these observations.

**Relevant high-risk area in Annex III of the EU AI Act:** 5(a) and/or 5(d) (Table 8)

**Table 8:** Analysis of use case 4: Flood mapping and monitoring

Parameters for analysis	High-risk scenario	Possible changes to lower the risk
Intended purpose	Identify areas to prioritize for flood relief supplies	Support preparedness for future hazard events
Objective	Determine level of damage caused by flood event	Identify flood-prone areas to plan mitigation measures
Context	Broad deployment over different regions	City- or neighborhood scale
Output	Ranking or Decision *e.g. rank areas from most to least urgent for flood relief or evacuation*	Prediction *e.g. identify flood-prone areas*
Degree of autonomy	Fully autonomous AI system	Flood maps are combined with other information to inform planning decisions
AI user	Governments	
AI subject	People	

The table above describes hypothetical examples of a high risk scenario and of a similar scenario that would actually not be high risk due to key differences. Here the scenario is high risk first because it falls within Annex III paragraph 5 (a) and/or (d); and second because of the following factors: (1) the intended purpose (identify areas to prioritize for relief) can engage a number of human rights notably because we may be dealing with life or death situations (see in-depth analysis below); (2) the output is a decision taken by the AI system, for example an order of priority; (3) the AI system is fully autonomous with no human involved. In the lower risk scenario, the intended purpose and objective are forward looking and not immediately connected to life or death scenarios. This already lowers the risk. However it does not eliminate it as the decisions based on the information collected may well lead to human rights impact down the line. In this use case as well the fact that the output is a mere prediction, and that other information is collected (i.e. the system is not fully autonomous) lowers the risk.

**In-depth analysis of fundamental rights and harm:**

This use case engages the right to non-discrimination (Article 21) since individuals may receive different levels of support depending on where they live. Due to monitoring of properties, Article 7 on the right to private and family life may also be infringed. This does not necessarily mean that the right is violated, as this right can be interfered with without violation under conditions outlined in Article 7(2) of the EU Charter. It could be, for example, that it is acceptable to monitor people’s homes to prevent disasters. Beyond ex ante monitoring, in the context of emergency response, the use of AI can affect two important rights: the right to life (Article 2) and the right to the integrity of the person (Article 3) if, for example, emergency services did not intervene in the right area leading to preventable deaths or injuries. Here the vulnerability of those affected may vary based on their socioeconomic status, health, age etc. The damage may be reversible or not depending on its extent (e.g. house contents damaged or entirely destroyed, injury, loss of life).

### A.5 Use case 5: drought monitoring for food security and health

**Description:** Monitoring droughts to optimize water usage and prevent or forecast crises and move from emergency support to development support. In some areas, especially in the Sahel region, droughts can starve isolated/neglected populations, force them to move, make them set up improvised camps, with health risks (e.g. cholera). Camps are supposedly temporary but may last years and have a devastating impact on the surrounding environment. Camps can also become ghettos if their occupants are unwanted. Detecting, or possibly even forecasting, droughts and managing them allows us to reduce these impacts or prevent them entirely.

**Relevant high-risk area in Annex III of the EU AI Act:** 5(a) and/or 5(d) (Table 9)

**Table 9:** Analysis of use case 5: Drought monitoring

Parameters for analysis	High-risk scenario	Possible changes to lower the risk
Intended purpose	Support food security and prevent food and health crises	Identify drought-prone areas to inform development efforts
Objective	Monitor droughts	Identify drought-prone areas
Context	Broad deployment over different regions; inhabited areas and agricultural land	Specific area; natural lands
Output	Decisions *e.g. requiring relocation out of drought-prone areas or controlling which crops can be planted*	Content *e.g. this area is experiencing water scarcity. Access to water is/is not possible*
Degree of autonomy	Fully autonomous AI System	Strong human in the loop requirement
AI user	Government agencies	
AI subject	Citizens	

The table above describes hypothetical examples of a high risk scenario and of a similar scenario that would actually not be high risk due to key differences. In the table, the scenario in the left column is high risk first because it falls within Annex III paragraph 5(a) and/or (d); and second because of the following factors: (1) the intended purpose is to support food security, which is a highly sensitive topic since food insecurity is linked to numerous human rights issues (see in-depth analysis below); (2) the output is a decision on issues that have life or death consequences (3) the AI system is fully autonomous with no human involved. By contrast, in the lower risk scenario, the intended purpose is to inform development efforts. While errors made by the AI system would be problematic, the facts that the output is “contents” and that humans would then interpret the data lower the risks significantly.

**In-depth analysis of fundamental rights and harm:**

This use case engages Article 21 on the right to non-discrimination. Like use case 4 above, people might be discriminated against based on where they live. Similarly, Article 7 on the right to private and family life may be infringed. Given the impact on food security and health, vulnerable individuals such as children (Article 24), the elderly (Article 25) and people with disabilities (Article 26) may be particularly impacted.

The harm can be economic in nature, for example of because of certain crops being restricted leading to decreased income. Harm can also be social harm due to enforced relocation. Irrespective of the nature of harm, the people impacted in this use case are typically vulnerable, due to a clear imbalance of power, knowledge, economic and social circumstances.

The harm can be partially reversible in case of economic harm, but would be irreversible in case of enforced displacement.

### A.6 Use case 6: detection of threatened tree species

**Description:** Companies that construct industrial buildings need to know in advance if there are some species of threatened plants, in particular trees, in an area where they are planning to realize new projects. These species are listed in the IUCN Red List, and usually are also protected under individual countries’ domestic law, though this is not necessarily the case. These companies are trying to use AI4EO to avoid, or limit, expensive surveys in the field.

**Relevant high-risk area in Annex III of the EU AI Act:** While this case is primarily about protecting plants and biodiversity, a use that is not explicitly recognized as high risk in the AI Act, it depends on the context. For example, for indigenous communities, land, plants and animals are closely connected to human rights, and it can be argued therefore that the use case concerns an AI system in the area of “Access to and enjoyment of essential private services and essential public services and benefits” (Annex III, paragraph 5(a)) (Table 10).

**Table 10:** Analysis of use case 6: Detection of threatened tree species

Parameters for analysis	High-risk scenario	Possible changes to lower the risk
Intended purpose	Providing project permits depending on the presence of threatened tree species	Identify threatened species for management purposes
Objective	Identify whether the number of endangered species within an area exceeds a threshold	Identify endangered species within an area
Context	Deployment on indigenous communities’ land	AI systems trained for specific endangered species relevant for a specific area
Output	Decision *e.g. whether construction is allowed*	Predictions *e.g. estimate presence of threatened species*
Degree of autonomy	High	Low
AI user	Governments	
AI subject	Companies	

The table above describes hypothetical examples of a high risk scenario and of a similar scenario that would actually not be high risk due to one key difference. In the table, the scenario in the left column is high risk first because it falls within Annex III paragraph 5(a) and/or (d); and second because the technology is deployed on indigenous land. Indigenous peoples have special rights that make the destruction of fauna and flora on their land a possible violation of their rights (see below).

**In-depth analysis of fundamental rights and harm:**

Indigenous peoples are not explicitly recognized and protected in the EU Charter for Fundamental Rights, but Article 7 (right to respect for private and family life), Article 21 (right to non-discrimination), Article 22 (Cultural, Religious, and Linguistic Diversity) and Article 37, read together, arguably protect these communities to the extent that their rights could be engaged if such an AI system was deployed. This would be especially the case if those articles were to be interpreted considering the international legal framework, especially the UN Declaration on the Rights of Indigenous Peoples.

In this example, the harm is primarily environmental in nature, and does not concern humans. If no humans are affected, then the use case is not regulated by the AI Act before harm to the environment is not covered. However, as mentioned above, for certain communities, environmental harm and human rights are closely connected. The use case also can lead to economic harm due to project delays or cancellation, and loss of reputation.

### A.7 Use case 7: air quality downscaling

**Description:** Air quality is observed by satellites and ground stations. Satellites have global coverage and sound the entire atmospheric column, but they have low spatial resolution and potentially high revisit times. Ground stations measure air pollution with the highest possible spatial and temporal resolutions, but they are few, sparse, and monitor only the lowest level of the atmosphere.

The downscaling of satellite observations to high resolution using AI models trained on ground station data is crucial for several reasons. It allows for a more detailed and accurate assessment of air quality, especially in urban areas where pollution levels can vary significantly over short distances. High-resolution air quality data is essential for identifying pollution hotspots, understanding local variations, and providing timely information for public health and environmental management. This fusion of satellite and ground-based data through AI enables a comprehensive and dynamic monitoring system for air quality, contributing to more effective pollution control and public awareness.

**Relevant high-risk area in Annex III of the EU AI Act:** 5(a) (Table 11)

**Table 11:** Analysis of use case 7: Air quality downscaling

Parameters for analysis	High-risk scenario	Possible changes to lower the risk
Intended purpose	Limiting pollution to ensure public health	Land use / infrastructure planning
Objective	Provide timely, spatial air quality levels to identify exceedance of norms	Predict effects of different infrastructure improvements on air quality in a city
Context	Broad deployment over different regions	City- or neighborhood scale
Output	Recommendation *e.g. where to implement measures*	Predictions *e.g. the potential effect of different planning scenarios*
Degree of autonomy	High	Low
AI user	Governments	
AI subject	Natural persons	

The table above describes hypothetical examples of a high risk scenario and of a similar scenario that would actually not be high risk due to key differences. In the table, the scenario in the left column is high risk first because it falls within Annex III paragraph 5(a); and second because of the following factors: (1) the intended purpose is to protect public health, which is a highly sensitive topic since poor public health decisions can lead to numerous human rights issues (see in-depth analysis below); (2) the output is a recommendation. Although this type of output is less controversial than a decision, a recommendation is still likely to be followed by the human(s) making the actual decisions; (3) the AI system has a high level of autonomy. By contrast, in the lower risk scenario, the intended purpose is improved land use and infrastructure planning. This in itself is probably enough to make the scenario low risk. In addition, the output is mere predictions and the system has a low degree of autonomy, which means that humans would then use the data in conjunction with other information.

**In-depth analysis of fundamental rights and harm:**

This case engages the right to environmental protection (Article 37) but also errors in the interpretation of readings of air quality will affect vulnerable individuals more than others. This means that Articles 21 (non-discrimination), 24 (children), 25 (elderly) and 26 (people with disability) are of relevance.

The harm can be economic in nature, industrial activities are limited and property value is thus lost if designated in a high-pollution zone. The harm can be to human health and also to the environment if pollution is underpredicted. Just like in the previous use case, this use case shows the limits of the AI Act. By focusing on fundamental rights and leaving aside environmental harm, the Act does not capture all ethical issues.

In this example harm can be reversed if it is purely economic, but health effects might be irreversible. Just like with the environmental harm example above, this use case shows the limitations of relying on the EU Charter for Fundamental Rights as the only legal benchmark for ethical conduct.

### A.8 Use case 8: mapping refugee camps

**Description:** The number of refugees worldwide is reaching unprecedented levels. The current large crises in the Near East and Ukraine increase the number to more than 120 million people forcibly displaced from their homes. International development institutions and governments may have projects to provide refugees with shelters and basic needs. However, the highly dynamic nature of conflict regions, the sheer number of planned and unplanned emergency shelters and their distribution across the globe often make it difficult to obtain adequate data and information on the current situation. Satellite, airplane, or drone data are thus oftentimes applied to assess the situations with basic cartographic data and indirectly to assess the number of people there and their living conditions. This data is also crucial when war situations or other conflict situations on the ground make it very dangerous or access is completely denied.

AI algorithms are being developed to automatically identify (i) infrastructure such as buildings, (ii) monitor the camps over time, (iii) assess the amount of people in the area, (iv) assess the living conditions, and (v) find access routes.

The basic goal is mapping the evolution of settlements for refugees or relocation camps. These are supposed to offer temporary shelters but often end up lasting many years. They are often designed for a limited number of people but end up hosting larger numbers. People there face hygienic hazards, with consequent health risks. They are exposed to natural hazards, such as floods, fires, and droughts. They often cause pollution in the form of garbage accumulation, water degradation, and smoke. Deforestation is often observed close to these camps. They suffer from insecurity in general. The guests of these camps suffer the consequences of these phenomena. Observing the evolution of these settlements allows the estimation of the number of people residing in these settlements and the status of the shelters.

**Relevant high-risk area in Annex III of the EU AI Act:** 2, 5(a, c, d), 7(b, d) (Table 12)

**Table 12:** Analysis of use case 9: Mapping refugee camps

Parameters	High-risk scenario	Possible changes to lower the risk
Intended purpose	Automate the delivery of supplies to refugee camps	Identify need of reserving more land for camp expansion
Objective	Automatically replenish supplies in refugee camps	Estimate camp expansion
Context	Broad deployment over different regions	Specific types of camps
Output	Decisions *e.g. identify which camps require more supplies*	Predictions *e.g. estimate growth of camp based on similar historical cases*
Degree of autonomy	Fully autonomous AI System	Human in the loop as experts interpret the results and combine with supplementary information
AI user	Governments/humanitarian aid	
AI subject	Refugees	

The table above describes hypothetical examples of a high risk scenario and of a similar scenario that would actually not be high risk due to key differences. Here the scenario is high risk first because it falls within Annex III paragraphs 2, 5(a, c, d), and 7(b, d); and second because of the following factors: (1) the intended purpose (automating the delivery of supplies) can engage a number of human rights notably because we may be dealing with life or death situations (see in-depth analysis below); (2) the output is a decision taken by the AI system, for example an order of priority; (3) the AI system is fully autonomous with no human involved. In the lower risk scenario, the intended purpose and objective are forward looking and not immediately connected to life or death scenarios. This already lowers the risk. However it does not eliminate it as the decisions based on the information collected may well lead to human rights impact down the line. In this use case the fact that the output is a mere prediction, and that other information is collected (i.e. the system is not fully autonomous) lowers the risk.

**In-depth analysis of fundamental rights and harm:**

This use case engages at least Article 1 (right to dignity), Article 7 (private and family life), Article 8 (data protection), Article 21 (non-discrimination), and Article 24 (rights of children). Arguably it also engages the rights of other especially vulnerable people besides children such as the elderly (Article 25) and people with disabilities (Article 26). Just like in use case 8, this use case can engage the right to an adequate standard of living, which includes the right to food, under Article 25 of the International Covenant on Economic, Social and Cultural Rights. But the right to food, just like the right to water, is not protected under the EU Charter of Fundamental Rights.

This use case concerns particularly vulnerable people who would have left their homes for safety reasons. The harm can be severe and irreversible especially for children who can suffer life long health issues because of food and clean water deprivation even for short periods of time.

### A.9 Use case 9: mapping informal settlements

**Description:** Large parts of the urban population in low- and middle-income countries (LMICs) live in informal settlements. These settlements are generally characterized by multiple levels of deprivation, including lack of adequate housing, lack of access to water and sanitation, high population densities, and high poverty rates.

International development institutions and governments may have projects to improve the infrastructure, and thereby the living conditions, of the residents. However, due to the unofficial nature of informal settlements and their often-rapid emergence, there is little data and knowledge of which infrastructure is present within them. Drones are sometimes used to map informal settlements to obtain this basic cartographic data, e.g. for disaster risk management purposes or urban planning interventions. For example, drones are being used by the city government of São Paulo, Brazil to identify risks of landslide and flooding in favelas. AI algorithms are being developed to automatically identify (i) infrastructure such as buildings, and (ii) elements which are considered to indicate elevated risk of landslides or flooding hazards.

Similar to the case of refugee camps, but here the main interest is in using satellite imagery to identify which areas are informal. That is, to put a label of formal vs informal to areas of the city, whereas the previous use case really focuses on identifying objects within the settlement. Automatically identifying informal settlements or slums is in high demand by, for example, international development agencies like the World Bank, yet the complexity of this task is often underestimated. Firstly, the appearance of slums differs greatly between cities, countries, and regions. It is therefore difficult to generate a representative training dataset for AI algorithms. Secondly, the characterization of slums is highly contextual. Experts often disagree and governments may wish to ignore the presence of slums. There is a conceptual ambiguity of informal settlements which will inevitably persist in AI models.

**Relevant high-risk area in Annex III of the EU AI Act:** 5(a, d) (Table 13)

**Table 13:** Analysis of use case 10: Mapping informal settlements

Parameters	High-risk scenario	Possible changes to lower the risk
Intended purpose	Flag informal growth for expropriation	Identify deprived areas for the planning of public services
Objective	Automatically identify informal settlements	Assess different degrees of deprivation of neighborhoods throughout a city
Context	Broad deployment over different regions	Informal settlements in a particular city
Output	Decisions *e.g. whether settlements should be expropriated*	Predictions *e.g. identify potential lack of services, levels of vulnerability*
Degree of autonomy	Fully autonomous AI System	Strong human in the loop requirement
AI user	Government	—
AI subject	People in informal settlements	—

The table above describes hypothetical examples of a high risk scenario and of a similar scenario that would actually not be high risk due to key differences. Here the scenario is high risk first because it falls within Annex III paragraph 5(a) and (d); and second because of the following factors: (1) the intended purpose (identifying areas for forced expropriation) can engage a number of human rights (see in-depth analysis below); (2) the output is a decision taken by the AI system ( = this area must be cleared of its informal buildings); (3) the AI system is fully autonomous with no human involved. By contrast, in the lower risk scenario, the intended purpose is to provide help by improving the living conditions of poorer neighborhoods. In addition the output is a mere prediction and humans remain involved in the actual decision.

**In-depth analysis of fundamental rights and harm:**

This use case engages at least Article 1 (right to dignity), Article 7 (private and family life), Article 8 (data protection), Article 16 (Freedom to conduct businesses), Article 21 (non-discrimination), and Article 24 (rights of children). Arguably it also engages the rights of other especially vulnerable people besides children such as the elderly (Article 25) and people with disabilities (Article 26). It also indirectly relates to environmental protection (Article 37) as these areas are often associated with bad environmental conditions.

In general, this use case concerns people who are economically vulnerable (Property, Article 17) and even short term economic harm could have severe, even irreversible consequences. Article 16 that protects the freedom to conduct a business may be violated. Stigmatization of an area that is mapped with a negatively co-notated semantic such as “informal”, “poor” or “slum” might negatively affect individual job opportunities, credit ratings or access to insurance. It thus brings a discrimination based on where someone lives and violates Article 21 on the right to non-discrimination. With it, Article 7 on the right to private and family life may also be infringed. Harm can also be social due to enforced relocation. If these areas are neglected by authorities, they will be under-equipped with social or technical infrastructure. And possibly also environmental protection (Article 37) is neglected. With it, this use case impacts security and health, and thus especially vulnerable individuals such as children (Article 24), the elderly (Article 25), and people with disabilities (Article 26). In general, the people potentially impacted in this use case are typically vulnerable. There is an imbalance of power, knowledge, and economic and social possibilities. Thus, as a result, acting persons have an uncanny responsibility.

## Footnotes

1 In this paper we refer to the full and final text of the EU AI Act as adopted on 13 June 2024. We consult the published text version from 12 July 2024, available here https://eur-lex.europa.eu/eli/reg/2024/1689/oj

2 The Charter of Fundamental Rights of the EU applies to EU institutions and member states. This document must be distinguished from the European Convention on Human Rights (ECHR), which applies to all 46 member states of the Council of Europe, which includes all EU member states as well as additional states from Eastern Europe and Central Asia, for example, Ukraine and Armenia.

3 The Explanatory Memorandum of the AI Act (COM(2021) 206 final; 2021/0106(COD) dated 21 April 2021) stated that the regulatory framework of the AI Act is proposed with the following objectives (p.3): (i) ensure that AI systems placed on the Union market and used are safe and respect existing law on fundamental rights and Union values; (ii) ensure legal certainty to facilitate investment and innovation in AI; (iii) enhance governance and effective enforcement of existing law on fundamental rights and safety requirements applicable to AI systems; (iv) facilitate the development of a single market for lawful, safe and trustworthy AI applications and prevent market fragmentation.

4 The regulation is expected to increase the costs associated with bringing a new AI System into the market. These costs will arise not only from the “conformity assessment fees” charged by the regulatory bodies (which are planned to be made affordable in the interest of SMEs – see Article 62) but also for compliance with and compilation of required regulatory processes and documentation.

5 This self-regulation is not permitted in the case of “high-risk” AI, which are heavily regulated by being subject to several mandatory provisions of the Act.

6 In an ideal world, providers of AI Systems (products or services) should also be encouraged to apply, on a voluntary basis, additional requirements related, for example, to environmental sustainability, accessibility to persons with disability, stakeholders’ participation in the design and development of AI systems, and diversity of the development teams. The Commission may develop initiatives, including of a sectoral nature, to facilitate the lowering of technical barriers hindering cross-border exchange of data for AI development, including on data access infrastructure, and semantic and technical interoperability of different types of data.
